# The Effect of Beta-Adrenergic Blocking Agents in Cutaneous Melanoma—A Nation-Wide Swedish Population-Based Retrospective Register Study

**DOI:** 10.3390/cancers12113228

**Published:** 2020-11-02

**Authors:** Dimitrios Katsarelias, Hanna Eriksson, Rasmus Mikiver, Isabelle Krakowski, Jonas A. Nilsson, Lars Ny, Roger Olofsson Bagge

**Affiliations:** 1 Department of Surgery, Institute of Clinical Sciences, Sahlgrenska Academy, University of Gothenburg, 405 30 Gothenburg, Sweden; dimitrios.katsarelias@gu.se (D.K.); jonas.a.nilsson@surgery.gu.se (J.A.N.); 2 Department of Surgery, Sahlgrenska University Hospital, 413 45 Gothenburg, Region Västra Götaland, Sweden; 3 Department of Oncology and Pathology, Karolinska Institutet, 171 64 Stockholm, Sweden; hanna.eriksson.4@ki.se (H.E.); isabelle.krakowski@sll.se (I.K.); 4 Theme Cancer/Department of Oncology, Karolinska University Hospital, 171 76 Stockholm, Sweden; 5 Regional Cancer Center South East Sweden and Department of Clinical and Experimental Medicine, Linköping University, 581 83 Linköping, Sweden; Rasmus.Mikiver@regionostergotland.se; 6 Theme Inflammation/Department of Dermatology, Karolinska University Hospital, 171 76 Stockholm, Sweden; 7 Department of Oncology, Institute of Clinical Science, Sahlgrenska Academy, University of Gothenburg, Sahlgrenska University Hospital, 405 30 Gothenburg, Sweden; lars.ny@oncology.gu.se; 8 Wallenberg Centre for Molecular and Translational Medicine, University of Gothenburg, 405 30 Gothenburg, Sweden

**Keywords:** melanoma, beta blocking agents, survival

## Abstract

**Simple Summary:**

Previous smaller studies have showed that a common heart medication, beta-blockers, potentially could reduce the risk of recurrence in patients with malignant melanoma and thereby increase survival. By combining different Swedish population-based registries, a total of 12,738 patients with melanoma were identified. Out of these patients 3702 had been prescribed beta-blockers and the remaining 9036 patients served as the control group. In a statistical analysis adjusting for known risk factors there was no effect of beta-blockers in reducing the risk of dying from melanoma. In conclusion, this population-based registry study could not verify the hypothesis that the use of beta blockers would improve survival in patients with melanoma.

**Abstract:**

Previous studies have demonstrated an anti-tumoral effect of beta-adrenergic blocking agents on cutaneous melanoma (CM). The aim of this study was to investigate if beta-adrenergic blocking agents have an impact on survival in Swedish patients with melanoma. A population-based retrospective registry study including all patients diagnosed with a primary invasive melanoma between 2009 and 2013 was performed. Data from the Swedish Melanoma Register were linked to the Swedish Prescribed Drug Registry and the Swedish Cause of Death Register. Cox regression analyses including competing risk assessments were performed. There were 12,738 patients included, out of which 3702 were exposed to beta-blockers vs. 9036 non-exposed patients. Age, male sex, Breslow thickness, ulceration, and nodal status were independent negative prognostic factors for melanoma-specific survival (MSS). Adding beta-blockers to the analysis did not add any prognostic value to the model (HR 1.00, *p* = 0.98), neither when adjusting for competing risks (HR 0.97, *p* = 0.61). When specifically analyzing the use of non-selective beta-blockers, the results were still without statistical significance (HR 0.76, *p* = 0.21). In conclusion, this population-based registry study could not verify that the use of beta-adrenergic blocking agents improve survival in patients with melanoma.

## 1. Introduction

Cutaneous melanoma (CM) has a rapidly increasing incidence, especially in the Nordic countries [1,2]. CM constitutes a major health problem and early detection and surgical removal of the primary tumor is crucial for the prognosis. Novel systemic therapies, targeting the MAP-kinase signaling pathway in patients carrying a *BRAF* V600 mutation or by modulating the immune response by means of checkpoint inhibitors, have dramatically improved survival in the metastatic setting of the disease [3,4,5]. There is an increasing research interest on agents that could potentiate the effect of currently used systemic treatments in the advanced disease.

A relatively new concept in this setting is so-called “drug repurposing”. The concept implies using well-established drugs for purposes different than their main indication and that may also be used in cancer treatment [6,7]. In particular, early preclinical observations and studies have shown a positive anti-tumoral effect of beta-adrenergic blocking agents (beta-blockers) on various tumors such as breast cancer, ovarian cancer, and melanoma [8,9,10,11,12,13]. Furthermore, preclinical studies demonstrate an anti-tumoral effect of propranolol on melanoma cells [8,14,15,16,17]. A newly published study showed that propranolol inhibits melanoma growth by both vasoconstriction and vasodilatation in a murine model [18]. There are also indirect effects of beta-adrenergic signaling by regulation of immunological factors important in antitumor response [19]. Recently, a prospective cohort study in patients with CM > 1 mm showed that propranolol protected against CM recurrence [20]. Although the number of patients enrolled was low, the results are promising and in conformity with the preclinical studies.

In Sweden, there are approximately 4000 new cases of primary invasive CM per year, and all patients are registered in the Swedish Melanoma Registry (SweMR) since 1990 with a coverage of more than 98%. We hypothesized that beta-blockers could have a positive impact on MSS. Therefore, we aimed to compare MSS between patients with CM receiving beta-blockers and patients not exposed to beta-blockers. We analyzed data from the SweMR and the Swedish Cause of Death Registry, a population-based registry with all death causes since 1952. We added data retrieved from the Swedish Prescribed Drug Registry (SPDR), with information on all dispensed drugs to Swedish patients since 1 July 2005.

## 2. Results

There were 12,738 patients included in the study and 6426 (50.5%) were women. The treatment group (beta-blockers) included 3702 patients. The median age for all the patients was 64 years. As expected, the median age was higher for the treatment group with 85% of the patients over the age of 60 compared to 50% in the control group. The median Breslow thickness was 0.9 mm and 18.9% had an ulcerated CM. Lymph node surgery, including sentinel node (SN) biopsy, and completion lymphadenectomy or dissection because of verified nodal metastases, was performed in 3317 patients. SNB was positive in 596 out of 2597 patients (22.9%). The demographic data of the population in the study according to the use of beta-blockers or not, is presented in Table 1.

In the multivariate Cox-regression analysis for MSS, significant independent prognostic factors were age, sex, Breslow thickness, ulceration, and nodal status (Table 2). Including the use of beta-blockers in the analysis did not add any significant prognostic value to the model (HR 1.00, CI 0.88–1.14, *p* = 0.98) and also when adjusting for competing risks the use of beta-blockers did not add any prognostic value (HR 0.97, CI 0.84–1.11, *p* = 0.61). We separately analyzed selective versus non-selective agents, and there was no statistically significant difference, although the non-selective group showed numerically better outcomes (HR 0.76, CI 0.50–1.16, *p* = 0.21), but without statistical significance (Table 2).

The multivariate Cox-regression analysis for OS, adjusted for age, sex, Breslow thickness, ulceration, and SN status, showed that in addition to the independent prognostic factors for MSS, the use of beta-blockers was a significant factor for worse outcomes in the treatment group with an HR of 1.25 (CI 1.16–1.34, *p* < 0.001). When performing an interaction test, it was shown that there was a significant interaction between age and selective beta-blockers, and when including this interaction in the analysis, the negative effect of selective beta-blockers was not significant any longer. The difference in HR from the univariate and the multivariate analysis for the use of selective beta-blockers (HR 2.58 and 1.28, respectively), is clearly indicative of possible residual confounding factors (Table 3).

The effect of the cumulative dose of beta-blockers was also studied, and the DDD for selective beta-blockers was dichotomized according to the median use (626.67 DDD). There was still no association concerning MSS between the use of beta-blockers and the control group (HR 0.93, CI 0.78–1.10, *p* = 0.406 for median > 626.67 DDD and HR 1.11, CI 0.95–1.31, *p* = 0.220 for median < 626.67 DDD).

## 3. Discussion

In this large national, population-based register study, the aim was to examine the effect of beta-blockers on survival for patients with CM. No statistically significant association between drug exposure and improved MSS was found. This applies for the entire study population who received the beta-blocker treatment and also for the subgroups analyzed according to the specific type of beta-blocker used (selective versus non-selective). There was also no correlation on the cumulative dose of beta-blockers, and when analyzing the DDD there was no correlation to MSS.

However, the non-selective beta-blockers subgroup analysis pointed towards a favorable MSS, although this was not statistically significant. Several published reports have shown a risk reduction in recurrence among CM patients receiving an adjuvant treatment with propranolol. De Giorgi et al. were the first to report a prolonged disease free survival (DFS), initially in a small series of patients with 2.5 years of follow-up [13], but also in a prospective off-label study [20]. In this later study, although a diminished risk for recurrence was shown, there was no statistically significant survival benefit in the group receiving propranolol, which is in accordance with our findings. Nevertheless, there were only six deaths due to CM in their cohort of 53 patients during the study period, and 14 recurrences in the control group. Although the study was underpowered, it resulted in an 80% risk reduction for relapse, advocating that prospective trials are necessary to conclude on this matter.

Recently published data from Wrobel et al. [21] showed the effect of non-selective wide range beta-blockers to be promising both in vitro and in a small series of patients, in terms of progression-free interval and MSS. The authors, however, point to the need for further prospective clinical trials that will investigate the role of non-selective beta-blockers as adjuvant to systemic melanoma therapies. Moreover, Kokolus et al. performed a retrospective study in 195 stage IV melanoma patients treated with immunotherapy. There were 65 patients that were prescribed beta-blockers for other conditions, parallel to their melanoma treatment and they found that patients with non-selective beta-blockers (pan-beta-blockers) had a statistically improved OS in comparison to those treated with selective beta-blockers and to those who had immunotherapy alone. They also showed tumor inhibition in a parallel murine model by the use of propranolol in addition to CTLA-4 and PD-1 inhibitors [22,23]. Since we did not include stage IV patients in our study, a beneficial role of pan-beta-blockers in addition to checkpoint inhibitors could not either be ruled out or confirmed by our results and thus, the above described findings deserve further attention and investigation. Following the publication of Kokolus et al., an ongoing prospective phase Ib/II trial investigates the potential role of propranolol when used together with pembrolizumab in patients with unresectable stage IIIC and IV melanoma (NCT03384836). This study is expected to complete recruitment by the end of 2020, and the results could probably help better define the role of non-selective beta-blockers for the treatment of melanoma in the new era of immunotherapy.

According to a recent meta-analysis by Yap A. et al., taking into consideration various tumor types including melanoma, there was no evidence that beta-blockers had any effect on recurrence or tumor specific survival [24]. Our results are also in accordance with previous registry-based studies by Livingston E. et al. in the Netherlands [25] and a similar study by McCourt et al. in the UK [26], that both failed to prove a survival benefit from the beta-blocker treatment among melanoma patients. However, a further analysis according to the type of beta-blocker used was only performed in the study by McCourt et al., which showed no impact on MSS [26]. A recent review study conducted by Ågesen et al. [27] tried to define the individual bioavailability of beta-blockers used in cardiology. A significant variation has been shown and this may be a confounding factor when trying to investigate the effect of the drugs in cancer patients, since dose adjustments and the timing of the treatment could be crucial when evaluating a possible favorable effect.

Our study has the advantage of being a population-based and nation-wide study based on data from high-quality registries with a high coverage rate with a low proportion of patients lost to follow-up. Not surprisingly, the treatment group had a worse OS, attributed to higher age and more co-morbidities, mainly cardiovascular, of the population in this group. We addressed this problem using a Fine-Gray risk model in order to adjust for this increased risk of death in the treatment group. Thus, the statistical analysis performed, which took into consideration not only competing risks but also adjusted for age and co-morbidities, can be considered reliable regarding the effect of beta-blockers on MSS for CM stages I–III.

Although the number of patients is large, the study is limited due to the retrospective design. In addition, the SPDR only supplies data on filled prescriptions, without taking into account if the patient actually took the drug as prescribed. A previous smaller series has mainly investigated recurrences and DFS, where DFS is used as an early surrogate marker for disease-specific survival. Unfortunately, reliable data concerning recurrences are lacking in most population-based registries and we were therefore not able to analyze recurrence as an endpoint. Potentially, there might be an improved effect of beta-blockers on DFS that we were unable to analyze, but this potential benefit did not translate into a benefit in MSS.

## 4. Materials and Methods

Data for all patients with a diagnosis of primary invasive CM (ICD C43.X) in Sweden between 1 January 2009 and 31 December 2013, were retrieved from the SweMR (*n* = 15,201). Patients with multiple CM (*n* = 1229), patients with advanced disease (stage IV) at the time of the initial diagnosis (*n* = 128), patients with missing information on either ulceration (*n* = 971) or Breslow thickness (*n* = 135) were excluded. Patients with stage IV disease where excluded since the known prognostic factors for this group of patients are different from patients with primary tumors. In total, 12,738 patients with a primary invasive CM (stage I–III) were eligible and were included in the final analysis.

Based on data from SPDR, there were 3702 patients that had been exposed to beta-blockers (both selective and non-selective) currently in use in Sweden (ATC code: C07) after the diagnosis of CM (treatment group) vs. 9036 patients without exposure to the beta-blocking treatment (control group). Data on the type of beta-blocker, prescription date, number of packages delivered, and defined daily doses (DDD) were also retrieved from the SPDR. The type of beta-blockers was divided into non-selective beta-blockers (C07AA) and selective beta-blockers (C07AB), patients on both selective BB and non-selective BB were defined as non-selective BB. DDD was defined as the assumed average maintenance dose per day when used for its main indication and it was dichotomized according to the median DDD for statistical analysis. DDD was used to analyze and adjust for the effect of exposure to the treatment where relations between the dose and response were explored.

Clinical and histopathological variables were retrieved from the SweMR including sex, age, and tumor thickness according to Breslow, ulceration status, and nodal status and were staged according to the American Joint Committee (AJCC) 8th edition [28]. Information on the date and cause of death were obtained from the Swedish Cause of Death Registry (SCDR), which was linked to SweMR to calculate MSS and OS. All patients were followed until death or to the end of follow-up on 20 Mars 2019, whichever came first. To minimize the risk for immortal time bias between the two groups, we calculated survival from the time of diagnosis.

The statistical analysis was performed using a multivariate Cox proportional hazard model for MSS and OS including the co-variates age, sex, Breslow thickness, ulceration, nodal status, exposure/non-exposure to beta-blockers, and the type of beta-blocker. A Fine-Gray competing risk model was used in order to verify the results adjusting for the increased risk of death, especially cardio-vascular disease related, in the beta-blocker exposed group (treatment group). All analyses were performed using R version 3.5.1 (The R Foundation for Statistical Computing).

The study was approved by the Regional Ethical Review Board at the University of Gothenburg (Dnr 11281/2018). The study was performed in accordance with the Declaration of Helsinki.

## 5. Conclusions

The impact of beta-blockers on melanoma survival is an interesting topic for further research, especially in the new era of novel systemic therapies that have dramatically changed the outcomes for patients with melanoma in stage IV. However, our findings do not support the hypothesis that beta-blockers would give a survival benefit for patients with cutaneous melanoma stage I–III, neither from the use of selective nor from non-selective beta-blockers. Nevertheless, given that previous studies have shown some positive effects, mainly for non-selective beta-blockers, a further investigation of these drugs is warranted, both in the preclinical and the clinical settings.

## Figures and Tables

**Table 1:** Patient and tumor characteristics in patients with primary stage I–III cutaneous melanoma diagnosed between 2009–2013 in Sweden.

Patient Characteristics	Beta-Blockers	No Beta-Blockers	*p*-Value
	(*n* = 3702)	(*n* = 9036)	
Age (median years)	73	60	<0.001
Age (years) (%)			<0.001
0–39	48 (1.3%)	1284 (14.2%)	
40–59	500 (13.5%)	3230 (35.7%)	
60–69	932 (25.2%)	2207 (24.4%)	
70–79	1141 (30.8%)	1369 (15.2%)	
>80	1081 (29.2%)	946 (10.5%)	
Gender *n* (%)			<0.001
Male	2070 (55.9%)	4242 (46.9%)	
Female	1632 (44.1%)	4794 (53.1%)	
Breslow (mm) *n* (%)			<0.001
≤1.0	1858 (50.2%)	5256 (58.2%)	
1.1–2.0	732 (19.8%)	1834 (20.3%)	
2.1–4.0	570 (15.4%)	1113 (12.3%)	
>4.0	542 (14.6%)	833 (9.2%)	
Ulceration *n* (%)			<0.001
Absent	2860 (77.3%)	7475 (82.7%)	
Present	842 (22.7%)	1561 (17.3%)	
Nodal surgery *n* (%)			0.002
None	2818 (76.1%)	6603 (73.1%)	
SLNB	687 (18.6%)	1910 (21.1%)	
Lymph node dissection	197 (5.3%)	523 (5.8%)	
Number of positive nodes			0.341
0	3505 (94.7%)	8512 (94.2%)	
1	115 (3.1%)	337 (3.7%)	
2–3	56 (1.5%)	132 (1.5%)	
>3	26 (0.7%)	55 (0.6%)	
Stage *n* (%)			<0.001
I	2417 (65.3%)	6615 (73.2%)	
II	1055 (28.5%)	1834 (20.3%)	
III	228 (6.2%)	583 (6.5%)	
Missing	2 (0.1%)	4 (<0.1%)	
Localization *n* (%)			<0.001
Trunk	1531 (41.4%)	3822 (42.3%)	
Lower extremity	690 (18.6%)	2142 (23.7%)	
Upper extremity	836 (22.6%)	1913 (21.2%)	
Head and neck	564 (15.2%)	996 (11.0%)	
Palmar/subungual	58 (1.6%)	108 (1.2%)	
Missing	23 (0.6%)	55 (0.6%)	
Mitoses *n* (%)			<0.001
Yes	1845 (49.8%)	4281 (47.4%)	
No	1124 (30.4%)	3119 (34.5%)	
Missing	733 (19.8%)	1636 (18.1%)	
Histological type *n* (%)			<0.001
SSM	2166 (58.5)	5976 (66.1)	
LMM	330 (8.9)	507 (5.6)	
NM	749 (20.2)	1386 (15.3)	
ALM	37 (1.0)	77 (0.9)	
Other	415 (11.2)	1086 (12.0)	
Missing	5 (0.1)	4 (<0.1)	

**Table 2:** Melanoma specific survival (MSS) and competing risks for the effect of beta-blockers in patients with primary stage I–III cutaneous melanoma diagnosed between 2009 and 2013 in Sweden.

Melanoma Specific Survival (MSS)			Cox-Regression				Competing Risk Model	
Patient Characteristics	Number of Patients	Number of Deaths (%)	Univariate	*p*-Value	Multivariate	*p*-Value	Multivariate	*p*-Value
			HR (95% CI)		HR (95% CI)		SHR (95% CI)	
Gender								
Men	6312	683 (10.8)	1		1		1	
Women	6426	419 (6.5)	0.57 (0.51–0.66)	<0.001	0.72 (0.64–0.81)	<0.001	0.73 (0.64–0.82)	<0.001
Age (years)	12,738	1102 (8.7)	1.04 (1.03–1.04)	<0.001	1.02 (1.02–1.03)	<0.001	1.01 (1.01–1.01)	<0.001
Breslow (mm)								
≤1.0	7114	99 (1.4)	1		1		1	
1.1–2.0	2566	186 (7.2)	5.43 (4.25–6.93)	<0.001	4.22 (3.30–5.41)	<0.001	4.24 (3.31–5.42)	<0.001
2.1–4.0	1683	342 (20.3)	17.42 (13.93–21.79)	<0.001	9.45 (7.44–12.22)	<0.001	9.23 (7.21–11.81)	<0.001
>4.0	1375	475 (34.5)	41.29 (33.23–51.31)	<0.001	16.93 (13.27–21.62)	<0.001	14.66 (11.32–18.98)	<0.001
Ulceration								
Absent	10,335	475 (4.6)	1		1		1	
Present	2403	627 (26.1)	7.46 (6.62–8.40)	<0.001	1.84 (1.61–2.11)	<0.001	1.72 (1.49–1.99)	<0.001
Number of positive nodes								
0	12,017	803 (6.7)	1		1		1	
1	452	156 (34.5)	6.20 (5.23–7.37)	<0.001	2.43 (2.03–2.90)	<0.001	2.60 (2.15–3.13)	<0.001
2–3	188	87 (46.3)	8.73 (7.00–10.89)	<0.001	2.99 (2.38–3.77)	<0.001	3.35 (2.62–4.29)	<0.001
>3	81	56 (69.1)	22.04 (16.78–28.94)	<0.001	6.73 (5.09–8.89)	<0.001	6.32 (4.42–9.05)	<0-001
Beta-blockers								
None	9036	717 (7.9)	1		1		1	
Selective	3251	385 (10.7)	1.51 (1.33–1.72)	<0.001	1.01 (0.88–1.15)	0.892	0.97 (0.84–1.12)	0.67
Non-selective	365	24 (6.6)	0.86 (0.57–1.29)	0.468	0.76 (0.50–1.14)	0.18	0.76 (0.50–1.16)	0.21
Other	86	13 (15.1)	2.00 (1.16–3.46)	0.013	1.32 (0.76–2.29)	0.324	1.52 (0.83–2.81)	0.18

**Table 3:** Overall survival (OS) in patients with stage I–III primary cutaneous melanoma diagnosed between 2009 and 2013 in Sweden.

Patient Characteristics	Number of Patients	Number of Deaths (%)	Univariate	*p*-Value	Multivariate	*p*-Value
			HR (95% CI)		HR (95% CI)	
Gender *n* (%)						
Men	6312	1864 (29.5)	1		1	
Women	6426	1348 (21)	0.67 (0.62–0.72)	<0.001	0.76 (0.71–0.81)	<0.001
Age (years)	12,738	3212 (25.2)	1.08 (1.08–1.09)	<0.001	1.07 (1.07–1.07)	<0.001
Breslow (mm) *n* (%)						
≤1.0	7114	950 (13.4)	1		1	
1.1–2.0	2566	580 (22.6)	1.77 (1.60–1.97)	<0.001	1.41 (1.27–1.56)	<0.001
2.1–4.0	1683	736 (43.7)	4.05 (3.68–4.46)	<0.001	2.08 (1.87–2.32)	<0.001
>4.0	1375	946 (68.8)	9.26 (8.46–10.14)	<0.001	3.30 (2.94–3.69)	<0.001
Ulceration *n* (%)						
Absent	10,335	1918 (18.6)	1		1	
Present	2403	1294 (53.8)	3.95 (3.68–4.24)	<0.001	1.49 (1.36–1.62)	<0.001
Number of positive nodes *n* (%)						
0	12,017	2837 (23.6)	1		1	
1	452	207 (45.8)	2.42 (2.10–2.79)	<0.001	1.87 (1.62–2.16)	<0.001
2-3	188	102 (54.3)	3.01 (2.47–3.67)	<0.001	2.16 (1.77–2.65)	<0.001
>3	81	66 (81.5)	8.03 (6.29–10.26)	<0.001	4.03 (3.15–5.17)	<0.001
Beta-blocker *n* (%)						
None	9036	1707 (18.9)	1		1	
Selective	3251	1384 (42.6)	2.58 (2.41–2.77)	<0.001	1.28 (1.19–1.38)	<0.001
Non-selective	365	95 (26.0)	1.44 (1.17–1.77)	<0.001	0.94 (0.76–1.16)	0.55
Other	86	26 (30.2)	1.74 (1.18–2.57)	<0.001	1.07 (0.72–1.57)	0.74
